# Gastric mixed neuroendocrine-non-neuroendocrine neoplasm (MiNEN) with pancreatic acinar differentiation: a case report

**DOI:** 10.1186/s13000-019-0815-3

**Published:** 2019-05-10

**Authors:** Yasuko Fujita, Noriyuki Uesugi, Ryo Sugimoto, Makoto Eizuka, Takayuki Matsumoto, Tamotsu Sugai

**Affiliations:** 1 Department of Molecular Diagnostic Pathology, School of Medicine, Iwate Medical University, 19-1 Uchimaru, Morioka, 020-8505 Japan; 2 Division of Gastroenterology, Department of Internal Medicine, School of Medicine, Iwate Medical University, Morioka, Japan

**Keywords:** Molecular analysis, BCL10, Stomach, MiNEN, Acinar-endocrine carcinoma

## Abstract

**Background:**

Gastric mixed neuroendocrine-non-neuroendocrine neoplasms (MiNENs) are infrequently encountered in routine practice. Some gastric neuroendocrine carcinomas (NECs) have a variety of differentiation patterns; however, pancreatic acinar differentiation in gastric NECs is rare. The molecular abnormalities of NECs with pancreatic acinar differentiation are not well understood.

**Case presentation:**

A 67-year-old male with a gastric MiNEN with pancreatic acinar differentiation without any symptoms. The tumor consisted of two components, including both glandular and solid histological features. Although the former component was a common type of adenocarcinoma, the latter showed endocrine differentiation and expressed pancreatic acinar enzymes immunohistochemically. A positive signal with the anti-BCL10 antibody, which detects one of the pancreatic acinar enzymes, was also present specifically in the latter component. We also examined *TP53* genomic mutations, DNA methylation status, and allelic imbalance (AI), which is an indicator of tumor aggressiveness. Although both components of this tumor showed no genomic mutation and a low methylation epigenotype, the frequency of AI was higher in the acinar-endocrine component than in the adenocarcinomatous component. The finding of AI indicated the progression of the conventional adenocarcinoma to an acinar-endocrine component and identified the aggressive potential of the acinar-endocrine component.

**Conclusions:**

We report a rare case of gastric MiNEN with pancreatic acinar differentiation. AI analysis revealed tumor progression and aggressiveness. In addition, the usefulness of the anti-BCL10 antibody for detecting the acinar-endocrine component was suggested.

## Background

Gastric cancer (GC) is comprised of various histological types. According to the World Health Organization (WHO) classification [1], GC is broadly classified into two histological subtypes, traditional carcinoma and neuroendocrine cell neoplasm (NEN). NEN is broadly sub-classified into neuroendocrine tumor (NET), neuroendocrine carcinoma (NEC) and mixed neuroendocrine-non-neuroendocrine neoplasm (MiNEN), which was previously termed “mixed adenoneuroendocrine carcinoma (MANEC)” [2]. The neuroendocrine component has a major role in determining the biological behavior of gastric MiNENs. Thus, it is important to identify the pathological and molecular characteristics of neuroendocrine carcinoma, namely “MiNEN”.

Some gastric NECs with a variety of other differentiation patterns, such as alpha fetoprotein-producing [3], squamous-cell [4] and pancreatic acinar-cell [5, 6] differentiation, have been reported to date. Among them, pancreatic acinar differentiation is of interest, given that such tumors are rare. In addition, although genetic events occurring in the tumor cells are essential for tumor development, the molecular abnormalities of NEC with pancreatic acinar differentiation are not well understood.

Here, we report a rare case of gastric MiNEN with pancreatic acinar cell differentiation and the results of an analysis of molecular alterations to identify the characteristics of this tumor.

## Case presentation

**Fig. 1:**
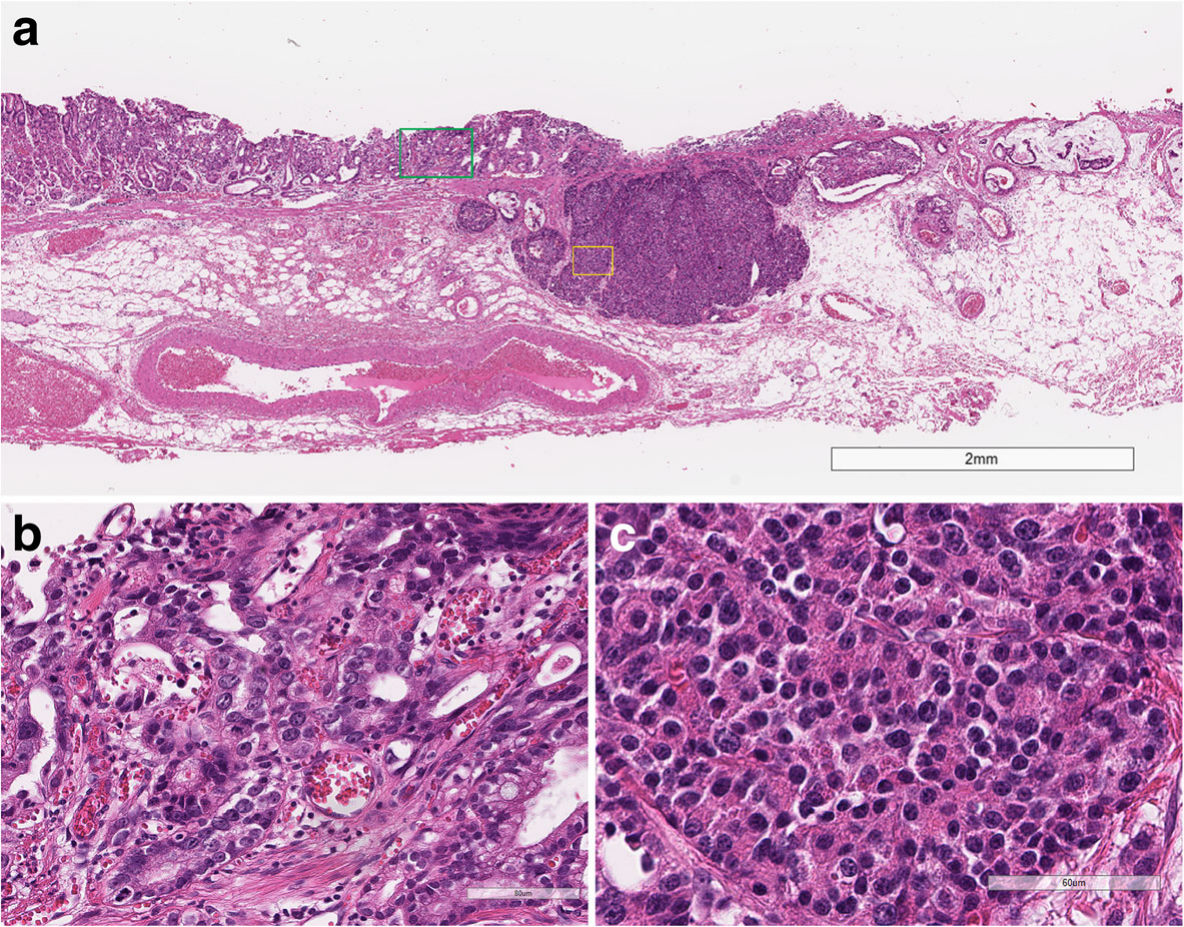
Histology of the tumor (hematoxylin and eosin [H&E] stain). **a** Low magnification. The green square indicates the area shown in (**b**), the yellow square indicates the area shown in (**c**). **b** The glandular tumor component. **c** The solid tumor component

A 67-year-old man underwent surveillance esophagogastroduodenoscopy once a year at our hospital after endoscopic submucosal dissection (ESD) for an early GC that was a conventional, well-differentiated tubular adenocarcinoma. He received eradication therapy for a week after the first ESD, after which he received no proton pump inhibitor medication. A surveillance endoscopy revealed another GC 3 years after the first ESD. No metastasis or primary tumor was detected in other organs, including the pancreas, by computed tomography. He underwent ESD for the new lesion, and the ESD specimen contained a 12 × 8 mm slightly depressed tumor with irregular margins. Histologically, the tumor was composed of two components, and it showed submucosal and lymphatic invasion (Fig. 1a). Although one component had a glandular structure and mucin production (Fig. 1b), the other had nested trabecular or acinar-like structures (Fig. 1c). The proportions of glandular and solid components were 60 and 40%, respectively. There were no ectopic pancreatic cells or pancreatic metaplasia in the background mucosa.

Because of the submucosal and lymphatic invasion, distal gastrectomy and lymphadenectomy were performed. There was no residual cancer in the surgically resected stomach and no lymph node metastasis. The patient has not received chemotherapy. He has remained alive without recurrence or metastasis for 15 months since ESD was performed.

## Immunohistochemical findings

**Fig. 2:**
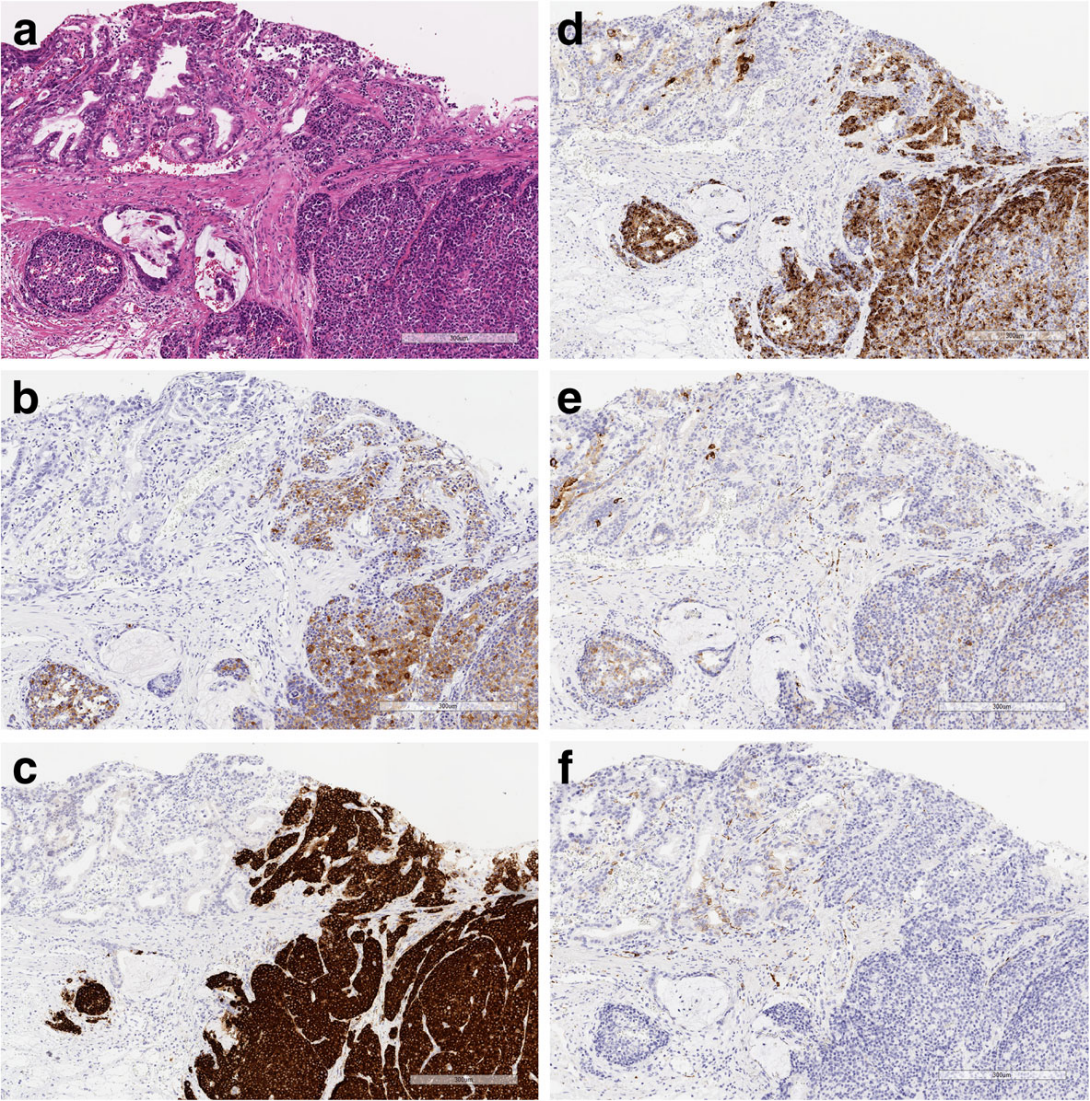
Immunohistochemical analysis of tumor components. **a** Hematoxylin and eosin (H&E)-stained specimen. **b**-**f** Immunohistochemical examinations. **b** Trypsin, **c** BCL10 and **d** Chromogranin A were positive in the solid component. **e** Synaptophysin and **f** CD56 were negative

Immunohistochemical studies were performed with representative sections (Fig. 2a) as previously described (Dako Envision system) [7]. The antibodies used are listed in Table 1. Each component was considered positive when more than 30% of the tumor cells were moderately or strongly stained. Although the glandular component was negative for trypsin (Fig. 2b), BCL10 (Fig. 2c) and chromogranin A (Fig. 2d), the solid component was positive for them. Although weak synaptophysin expression was observed in the solid component (Fig. 2e), it was considered negative. CD56 was not expressed in either component (Fig. 2f).

**Table 1:** Results of immunohistochemical analysis

Antibody (clone)	M/P	Manufacturer	Dilution	Glandular component	Solid (acinar-endocrine) component
Trypsin	M	EMD Millipore	1:40000	–	+
BCL10 (331.3)	M	Santa Cruz Biotechnology	1:200	–	+
Chromogranin A	P	Agilent technologies	RTU	–	+
Synaptophysin (DAK-SYNAP)	M	Agilent technologies	RTU	–	–
CD56 (123C3)	M	Agilent technologies	RTU	–	–

*M* monoclonal antibody, *P* polyclonal antibody, *−* negative, *+* positive, *RTU* ready-to-use

The immunohistochemical results are summarized in Table 1.

## Tissue dissection and DNA extraction

DNA from each component was extracted from stereoscopically dissected paraffin-embedded tissue sliced at a 10-μm thickness, and including more than 60% of tumor cells, with TaKaRa DEXPAT (TAKARA Bio Inc., Japan) according to the manufacturer’s instructions.

## Mutation analysis of the *TP53*, *KRAS*, *BRAF* and *GNAS* genes

The *TP53* gene (exons 5 to 8) was analyzed with polymerase chain reaction single-strand conformation polymorphism (PCR-SSCP) analysis followed by PCR direct sequencing as described previously [8]. No mutation was found in either of the tumor components.

## Microsatellite analysis

**Fig. 3:**
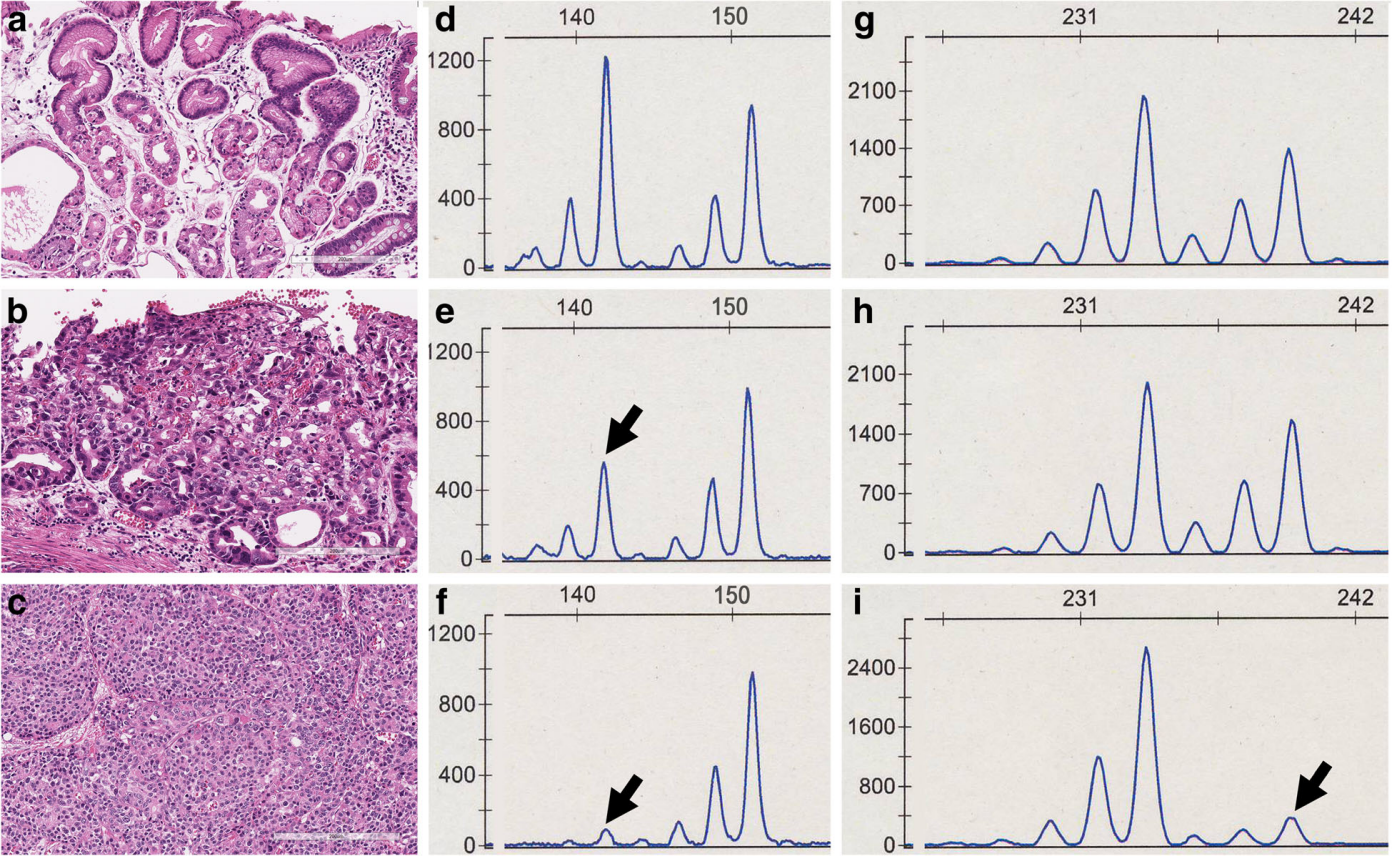
Representative results of allelic imbalance analysis. **a**-**c** Consecutive hematoxylin and eosin (H&E)-stained specimen of the tissue sampled for DNA extraction. **a** Non-neoplastic mucosa sampled for **d** and **g**, **b** Glandular tumor component sampled for **e** and **h**, and **c** Acinar-endocrine component sampled for **f** and **i**. **d**-**f** The alleles at D8S532. **e** and **f** show loss of heterozygosity (LOH) (black arrows). **g**-**i** The alleles at D11S5014. **h** showed heterozygosity, but **i** showed LOH (black arrow)

Allelic imbalance (AI) was examined to determine the aggressiveness of the solid component with a PCR-microsatellite assay (GeneAmp PCR System 9600; Perkin-Elmer, CA, USA) according to previously reported procedures [7]. AI on chromosomes 1p, 5q, 8p, 11, 18p and 22q was examined with 27 highly pleomorphic microsatellite markers often associated with AI in GCs, shown in Table 2. The results of the AI analysis are also shown in Table 2. Although AI was detected on chromosomes 5q, 8p, 11q and 22q in the solid component, AI was detected only on chromosome 11q in the glandular component (Figs. 3a-i).

**Table 2:** Results of allelic imbalance analyses

Markers	Location	Glandular component	Acinar-endocrine component
D1S228	1p36.13	H	H
D1S548	1p36.31-p36.23	N	N
D5S107	5q14.3	H	LOH
D5S299	5q15-q22	H	LOH
D5S82	5q21.3	H	LOH
D5S346	5q22.2	H	H
D8S513	8p11	N	N
D8S532	8p12	H	LOH
D8S201	8p23.2	H	LOH
D11S576	11p15.5	N	N
D11S922	11p15.5	H	H
D11S1318	11p15.5	N	N
D11S5011	11q23.1	N	N
D11S5014	11q23.1	LOH	LOH
D11S5015	11q23.1	LOH	LOH
D11S5017	11q23.1	N	N
D11S5018	11q23.1	N	N
D11S5019	11q23.1	N	N
D11S912	11q24.3	H	LOH
D11S969	11q25	LOH	LOH
D11S1320	11q25	H	N
D18S34	18q12	H	H
D18S487	18q21.2	H	H
DCC	18q21.2	H	H
D22S1140	22q13	H	H
D22S1168	22q13	H	LOH
D22S274	22q13	H	LOH

*N* not informative, *H* heterozygosity, *LOH* loss of heterozygosity

## DNA methylation analysis

DNA methylation status was classified as low, intermediate or high, with a two-step method [9]. The cutoff value was more than 30% of the tumor. In this case, the DNA methylation status of each component was determined to be a low methylation epigenotype.

## Discussion and conclusions

Gastric MiNEN is an uncommon tumor, and gastric MiNEN with pancreatic acinar differentiation is extremely rare. The present GC was composed of conventional adenocarcinomatous and solid-acinar differentiated components. Although NEN is generally diagnosed by immunohistochemical expression of at least two of three markers, chromogranin A, synaptophysin and CD56, the solid component of the present tumor was positive only for chromogranin A, and showed faint expression of synaptophysin that was considered negative. Different general markers for identifying neuroendocrine differentiation are used in different organs [10]. In the gastrointestinal tract, chromogranins and/or synaptophysin are used [11]. In addition, the definition of pancreatic neuroendocrine carcinoma by the WHO classification is described relative to markers of neuroendocrine differentiation as “diffuse or faint synaptophysin and faint or focal chromogranin A staining” [12]. Therefore, the solid component with pancreatic acinar differentiation can be considered as an endocrine carcinoma component.

To the best of our knowledge, only four cases of GC with ductal, endocrine and pancreatic acinar differentiation have been reported to date [5, 6]. Although one of the four reported cases had shown an elevated serum lipase, the others and the present case showed no specific serum markers. Among the reported cases, two had lymph node metastases, and one also had a liver metastasis. In the present case, lymphatic invasion was prominent. These findings suggest the clinically aggressive behavior of this tumor.

We examined the genetic alterations of this MiNEN with pancreatic differentiation to determine the pathogenesis and aggressiveness of this tumor. Although multiple AIs were found in the NEC component, AI on 11q was commonly observed in both components. Although mutation analysis is necessary to confirm that different components within the same tumor have the same origin, AI analysis may be helpful to clarify the origin [13]. This finding suggests that the NEC component arose from adenocarcinomatous cells with 11q AI. Second, the finding that multiple areas of AI occurred in the NEC component suggests that the NEC component acquired more aggressiveness compared with the adenocarcinomatous component. It is well known from previous studies that multiple areas of AI result from a more aggressive potential of the tumor cells [14]. This finding suggests that AI analysis may be useful to predict the aggressiveness of tumor cells.

In addition, some reports have described molecular analyses of carcinomas with acinar differentiation of the pancreas [15, 16]. Jiao et al. showed frequent loss of heterozygosity (LOH) of chromosome 11p (52%) and 18q (57%) [15]. Although LOH of chromosome 11p was reported in acinar cell carcinoma of the pancreas, it was not found in the present tumor. Therefore, the tumorigenesis of the present tumor may not be similar to pancreatic acinar carcinoma. On the other hand, Bergmann et al. examined four mixed acinar-neuroendocrine carcinomas by comparative genomic hybridization and reported gains of 1q, 5, 4p, 7, 12q, 13, 16, 17q, 20 and losses of 1p, 5q, 8p, 9p, 11q, 13q, 16q, 18q [15]. AIs on some of those loci were found in the present tumor.

Previous data showed that epigenetic alterations, such as DNA methylation, are actively involved in changes in gene expression as a result of tumor development [17]. In a recent study, it was shown that DNA methylation is prevalent in GC development [18]. DNA methylation may serve as a useful marker that may identify a distinct subset of GC. In the present study, however, a low level of DNA methylation was found within the two components. This finding suggests that accumulation of DNA methylation was not actively involved in the progression of this tumor.

Previous studies have shown that mutation of the *TP53* gene has a major role in the progression of GC [17, 19]. Such mutation contributes to tumor development in NEC [20]. In the present study, however, no *TP53* mutation was found in either component. Although only exons 5 to 8 of the *TP53* gene, which is the hot spot for mutation in gastrointestinal cancers, were examined in this study, overexpression of TP53 was not observed in either component immunohistochemically. This finding may suggest that *TP53* mutation is not closely associated with the carcinogenesis of this MiNEN.

Immunohistochemical analysis with an anti-BCL10 antibody was very useful for detecting the aggressive component of this tumor by identifying the pancreatic differentiation of acinar cells in the tumor. Although other anti-pancreatic enzyme antibodies, including those against trypsin and amylase, have also been used to show pancreatic acinar differentiation, the monoclonal anti-BCL10 antibody is known as a sensitive and specific marker of pancreatic acinar cells or acinar differentiation [21]. In the present case, anti-BCL10 antibody staining was more strongly and specifically positive for the acinar-differentiated component than anti-trypsin antibody staining. These findings indicate that the anti-BCL10 antibody is a valuable tool to identify aggressive acinar-endocrine components of GCs.

In conclusion, we report a case of gastric MiNEN with pancreatic acinar differentiation. This is the first report to confirm the progression of a conventional adenocarcinoma to an acinar-endocrine component and to assess the aggressive potential of an acinar-endocrine component by AI analysis. In addition, our case indicates the usefulness of anti-BCL10 antibody staining for detecting aggressive acinar-endocrine components.

## Abbreviations

AI: Allelic imbalance
ESD: Endoscopic submucosal dissection
GC: Gastric cancer
MANEC: Mixed adenoneuroendocrine carcinoma
MiNEN: Mixed neuroendocrine-non-neuroendocrine neoplasm
NEC: Neuroendocrine carcinoma
NEN: Neuroendocrine cell neoplasm
NET: Neuroendocrine tumor
PCR-SSCP: Polymerase chain reaction single-strand conformation polymorphism
WHO: World Health Organization
